# A Novel Feature Extraction Method for Power Transformer Vibration Signal Based on CEEMDAN and Multi-Scale Dispersion Entropy

**DOI:** 10.3390/e23101319

**Published:** 2021-10-09

**Authors:** Haikun Shang, Junyan Xu, Yucai Li, Wei Lin, Jinjuan Wang

**Affiliations:** Key Laboratory of Modern Power System Simulation and Control & Renewable Energy Technology, Ministry of Education, Northeast Electric Power University, Jilin 132012, China; 2201900164@neepu.edu.cn (J.X.); 2201900107@neepu.edu.cn (Y.L.); 2202000096@neepu.edu.cn (W.L.); 2202000007@neepu.edu.cn (J.W.)

**Keywords:** CEEMDAN, MDE, DPC, power transformer, vibration signal, feature extraction

## Abstract

Effective diagnosis of vibration fault is of practical significance to ensure the safe and stable operation of power transformers. Aiming at the traditional problems of transformer vibration fault diagnosis, a novel feature extraction method based on complete ensemble empirical mode decomposition with adaptive noise (CEEMDAN) and multi-scale dispersion entropy (MDE) was proposed. In this paper, CEEMDAN method is used to decompose the original transformer vibration signal. Additionally, then MDE is used to capture multi-scale fault features in the decomposed intrinsic mode functions (IMFs). Next, the principal component analysis (PCA) method is employed to reduce the feature dimension and extract the effective information in vibration signals. Finally, the simplified features are sent into density peak clustering (DPC) to get the fault diagnosis results. The experimental data analysis shows that CEEMDAN-MDE can effectively extract the information of the original vibration signals and DPC can accurately diagnose the types of transformer faults. By comparing different algorithms, the practicability and superiority of this proposed method are verified.

## 1. Introduction

Transformers play an important role in the power system. Their safe operation directly affects the reliability and economy of power supply [1]. It is of great significance to study the fault detection technology of power transformers. The recent research shows that the vibration of transformers is strongly related to the mechanical properties of windings [2]. Therefore, more attentions have been paid to transformers vibration signal analysis [3,4].

The impact of different vibration faults on transformers is obviously different. Therefore, effective identification of different vibration faults is important for transformers. As different vibration faults have diverse signal characteristics [5], feature extraction plays a key role in the process of transformer vibration fault recognition, which can directly affect the recognition results. At present, a large number of scholars have paid more attention to transformer vibration fault analysis and achieved extensive gratifying results [6,7]. Berler et al. analyzed the surface vibration signal of no-load and load transformers by fast Fourier transform. However, this study ignored the influence of different loading conditions on winding vibration signals [8]. Zhao proposed an approach based on cross wavelet analysis to obtain the amplitude and frequency characteristics of transformer vibration. However, it is difficult to form a certain criterion to measure the degree of power transformer faults [9]. Huang proposed an adaptive signal processing method named empirical mode decomposition (EMD), which can decompose a complex signal into finite intrinsic mode functions (IMFs) and a residual component [10]. EMD has been widely applied to the processing of many non-linear signals [11,12,13]. However, EMD is limited due to the inherent problems of mode mixing. In order to eliminate the mode mixing phenomenon in EMD, N.E. Huang proposed ensemble empirical mode decomposition (EEMD) [14]. In EEMD decomposition, the Gaussian white noise is added into original signals. The noise is eliminated by the repeated averaging method. It can effectively make up for the lack of signal scale. EEMD is also suitable for non-linear and non-stationary signals. It has been widely applied to feature extraction in recent years [15,16,17]. Zhao et al. used EEMD method to extract the characteristics of vibration signal in the case of transformer winding fault [18]. As EEMD does not isolate the residual noise, the added white noise can be transferred from high frequency to low frequency. Therefore, a certain amount of noise will be retained in the IMFs, which will affect the subsequent results of signal processing. In order to overcome the shortcomings of EEMD and improve the completeness of decomposition, Torres et al. proposed complete ensemble empirical mode decomposition with adaptive noise (CEEMDAN). In this method, adaptive noise is added to the signal decomposition. By changing the decomposition process, the residual noise in IMFs can be reduced. CEEMDAN can improve the operation efficiency and retain the information of the original signal [19]. It is nowadays used in many different research areas. In reference [20], a morphological structure of ECG signals based on CEEMDAN algorithm was proposed. Experimental results show that the proposed de-noising method can eliminate both high-frequency interference and low-frequency baseline drift. It shows better performance compared with other de-noising methods. In [21], CEEMDAN was employed for feature extraction of reciprocating machinery. This method can effectively extract the fault features of reciprocating pump vibration signal, and the test patterns can be accurately identified with the established classification model. Reference [22] proposed a new fault prediction method for rotating machinery based on CEEMDAN. The results demonstrate that this approach is superior to other methods. However, the application of CEEMDAN in the transformer vibration signal has not been found yet. Therefore, CEEMDAN will be a useful attempt for transformer vibration feature extraction in this work.

Entropy was introduced by Shannon as a measurement of uncertainty or irregularity [23]. Entropy can effectively quantify the complexity and uncertainty of time series. It has been widely used in mechanical fault diagnosis. Sample entropy (SE) has been widely used in signal and image processing [24]. However, it is not fast enough for long signal applications. Permutation entropy (PE) is fast in calculation [25]. However, it does not consider the difference between the average and original amplitude [26]. Considering this, Rostaghi and Azami proposed dispersion entropy (DE) [27]. DE is better than PE method in distinguishing different groups of each dataset. In addition, the calculation time of DE is significantly less than that of SE and PE. References [28] and [29] have verified the effectiveness of DE in mechanical fault diagnosis. According to the concept of multi-scale analysis, Azami et al. proposed multi-scale dispersion entropy (MDE), which can overcome the defects of signal estimation at one single scale [30]. In this paper, MDE will be used to quantify the characteristics of transformer vibration signals.

Density peaks clustering (DPC) is an unsupervised clustering learning method, which can automatically find the correct number of clusters without presetting [31]. In reference [32], the adaptive DPC algorithm was used to select the cutoff distance to identify different bearing and gear faults. In reference [33], a bearing fault diagnosis method based on DPC was proposed. It shows that the method can accurately diagnose the bearing fault. Above research demonstrates that DPC method is suitable for fault diagnosis in the practical engineering. In this paper, DPC is used to cluster the feature vectors extracted from the vibration signal to identify the transformer faults.

In this work, a novel feature extraction method based on CEEMDAN and MDE is proposed for power transformers vibration signal. DPC is employed for fault diagnosis with extracted features. The simulation and experimental signals verify the effectiveness of the proposed method.

The rest of this paper is organized as follows: Section 2 introduces the conception of CEEMDAN, MDE, and DPC, and also presents the steps of fault diagnosis for transformers vibration signal based on CEEMDAN, MDE, and DPC. In Section 3, the simulation analysis and the experimental results based on the proposed method are stated. Additionally, then the performance of the proposed method is compared with different methods. Finally, Section 4 concludes this paper.

## 2. Fault Diagnosis for Vibration Signal of Transformers Based on CEEMDAN-MDE and DPC

### 2.1. CEEMDAN Principle

CEEMDAN is an improved method based on EEMD proposed by Torres [19]. The decomposition principle of CEEMDAN is as follows:

Let $E_{j}\left(\cdot \right)$ be the *j^th^* IMF obtained by EMD decomposition, and the *j^th^* IMF obtained by CEEMDAN decomposition be $\bar{IMF_{j}\left(t\right)}$. The decomposition steps of CEEMDAN are as follows:

1. A new signal $x\left(t\right)+\varepsilon_{0}w^{i}\left(t\right)$ is obtained by adding positive and negative paired Gaussian white noise to *x*(*t*). EMD is used to decompose the new signal to obtain *IMF*_1_.
  (1) $$E\left(x\left(t\right)+\varepsilon_{0}w^{i}\right)=IMF_{1}^{i}\left(t\right){+r}^{i}$$
  where E(⋅) represents EMD decomposition, $w^{i}$ represents the Gaussian white noise signal satisfying the standard normal distribution, $\varepsilon_{0}$ represents the standard deviation of the noise, *x*(*t*) represents the original signal, $IMF_{1}^{i}\left(t\right)$ represents the *IMF*_1_ component decomposed by EMD, and $r^{i}$ represents the first residual component;
2. By averaging $IMF_{1}^{i}\left(t\right)$, the first component of IMF can be obtained in Formula (2).
  (2) $$\bar{IMF_{1}\left(t\right)}=\frac{1}{N}\sum_{i=1}^{N}IMF_{1}{}^{i}\left(t\right)$$
3. Calculate the first residual component *r*(*t*).
  (3) $$r_{1}\left(t\right)=x\left(t\right)-\bar{IMF_{1}\left(t\right)}$$
4. A new signal is obtained by adding a pair of positive and negative Gaussian white noise to *r*_1_(*t*), and *IMF*_1_ is obtained by EMD decomposition. Calculate *IMF*_2_.
  (4) $$\bar{IMF_{2}\left(t\right)}=\frac{1}{N}\sum_{i=1}^{N}E_{1}\left(r_{1}\left(t\right)+\varepsilon_{1}E_{1}\left(w^{i}\left(t\right)\right)\right)$$
5. Calculate the second residual component *r*_2_(*t*).
  (5) $$r_{2}\left(t\right)=r_{1}\left(t\right)-\bar{IMF_{2}\left(t\right)}$$
6. Repeat the above steps until the residual signal is a monotone function and cannot be further decomposed. When the number of eigenmode components is *k*, the original signal *x*(*t*) is decomposed as:
  (6) $$x\left(t\right)=\sum_{k=1}^{K}\bar{IMF_{k}\left(t\right)}+r_{k}\left(t\right)$$

### 2.2. Multi-Scale Dispersion Entropy

DE is an algorithm for measuring the complexity of time series [27]. DE can effectively solve the problem of long-time calculation in SE. Moreover, it fully considers the difference between amplitudes which cannot be realized in PE. In order to quantify the complexity of multivariate time series, MDE is introduced. The detailed steps are as follows:

1. A multivariable time series is mapped from $X={\left\{x_{k,i}\right\}}_{k=1,2,\ldots ,n}^{i=1,2,\ldots ,N}$ to $Y={\left\{y_{k,i}\right\}}_{k=1,2,\ldots ,n}^{i=1,2,\ldots ,N}$ by Formula (7).
  (7) $$y_{k,i}=\frac{1}{\sigma_{k}\sqrt{2\pi }}\overset{x_{k,i}}{\underset{-\infty }{\int }}e^{\frac{-{\left(t-\mu_{k}\right)}^{2}}{2\sigma_{k}^{2}}}dt$$
  where *µ* represents the expectation, and *σ^2^* represents the variance;
2. *Y* is mapped to $Z={\left\{z_{k,i}\right\}}_{k=1,2,\ldots ,n}^{i=1,2,\ldots ,N}$ (from 1 to *c*) by linear transformation.
  (8) $$z_{k,i}=round\left(c_{\cdot }y_{k,i}+0.5\right)$$
  where *c* represents the class, *Z_k,i_* shows the *j^th^* member of the classified time series and rounding involves either increasing or decreasing a number to the next digit;
3. According to the multi-scale embedding theory, the time series *Z_m_* is reconstructed as follows.
  (9) $$Z_{m}\left(j\right)=\left[z_{1,j},z_{1,j+d_{1}},\ldots ,z_{1,j+\left(m_{1}-1\right)d_{1}},\ldots ,z_{n,j},z_{n,j+d_{n}},\ldots ,z_{n,j+\left(m_{n}-1\right)d_{n}}\right]$$
  where $j\in [1,N-\left(m-1\right)d],m=\left[m_{1},m_{2},\ldots ,m_{n}\right]$ represents the embedding dimension;
4. All combinations of m elements in each *Z_m_*(*j*) are created, named $\phi_{q,l}\left(j\right)$. Additionally, each $\phi_{q,l}\left(j\right)$ is mapped to dispersion patterns $\psi_{v_{0}v_{1}\ldots v_{m-1}}\left(v=1,2,\ldots ,c\right)$;
5. The probability of each dispersion patterns can be calculated by Equation (10).
  (10) $$p\left(\psi_{v_{0}v_{1}\ldots v_{m-1}}\right)=\frac{Number\left(\psi_{v_{0}v_{1}\ldots v_{m-1}}\right)}{[N-\left(m-1\right)d]C_{m}^{mn}}$$
  where $Number\left(\psi_{v_{0}v_{1}\ldots v_{m-1}}\right)$ represents the number of the $\psi_{v_{0}v_{1}\ldots v_{m-1}}$ in the $\phi_{q,l}\left(j\right)$, d represents the time delay. $C_{m}^{mn}$ represents total number of combinations per *Z_m_*(*j*);
6. Finally, according to the definition of Shannon entropy, the MDE of multi-scale data *X* is as follows.
  (11) $$MvDE\left(x,m,c,d\right)=-\sum_{\psi =1}^{c^{m}}p\left(\psi_{v_{o}v_{1}\cdots v_{m-1}}\right)\ln\left[p\left(\psi_{v_{o}v_{1}\cdots v_{m-1}}\right)\right]$$

### 2.3. Density Peaks Clustering

DPC was proposed by Alex Rodriguez and Alessandro Laio. This algorithm is mainly based on the local density of data points. The principle of DPC is simple and clear. It is not sensitive to outliers [31].

In datasets $X=\left\{x_{1},x_{2},\cdots ,x_{N}\right\}$, $x={\left\{x_{i1},x_{i2},\cdots ,x_{ip}\right\}}^{T}$, *X_ij_* represent the *j^th^* dimension attribute value of the *i^th^* data point. DPC algorithm only needs to calculate two variables: the local density of sample points *ρ* and the distance *δ* between the point and the nearest neighbor with higher density. For each data point *x_i_*, the formula of local density *ρ* is as follows.

(12) $$\rho_{i}=\sum_{j,j\neq i}\chi \left(d_{ij}-d_{c}\right)$$

where χ represents the Characteristic function, $d_{ij}=dist\left(x_{i},x_{j}\right)$ represents a certain distance between two data points and *d_c_* represents a cutoff distance.

The distance of the point *x_i_* is defined as:

(13) $$\delta_{i}=\{\begin{array}{c} \underset{j:p_{j}>p_{i}}{\min}\left(d_{ij}\right)\begin{array}{cc} & \rho_{i}<\max\left(\rho \right) \end{array}\\ \underset{j}{\max}\left(d_{ij}\right)\begin{array}{cc} & \rho_{i}=\max\left(\rho \right) \end{array} \end{array}$$

In order to determine the number of clustering centers, DPC algorithm also needs to calculate the number of each sample point *γ.*

(14) $$\gamma_{i}=\rho_{i}\delta_{i}$$

With local density *ρ_i_* and the distance $\delta_{i}$ of all points, the decision graph can be drawn. In the decision graph, the points whose *ρ_i_* and $\delta_{i}$ are larger will be selected as the cluster center. The remaining points will be attributed to the cluster of the nearest neighbor with higher density. Additionally, then the final clustering result will be obtained.

### 2.4. Steps of Fault Diagnosis for Transformers Vibration Signal

In this work, CEEMDAN and MDE are employed for transformer vibration feature extraction. Firstly, the CEEMDAN method is used to decompose the original transformer vibration signal, and the correlation coefficient (*CC*) is applied to the selection of IMF components. Secondly, the MDE of IMF components are calculated and the eigenvalue matrix is formed. Then, PCA method is used to reduce the dimension of the eigenvalue matrix. Finally, DPC is used to cluster the extracted eigenvalues. The specific steps are as follows:

1. Transformer vibration signals are extracted in the laboratory under different working conditions;
2. The extracted vibration signals are decomposed by CEEMDAN method and a series of IMF components are obtained;
3. In this paper, to extract useful and effective IMF components, *CC* analysis is employed for IMF selection [34]. *CC* represents the correlation between signals, defined in Formula (15).
  (15) $$CC=\frac{\sum_{i=1}^{n}\left(r_{i}-\bar{r}\right)\left(x_{i}-\bar{x}\right)}{\sqrt{\sum_{i=1}^{n}{\left(r_{i}-\bar{r}\right)}^{2}}\sqrt{\sum_{i=1}^{n}{\left(x_{i}-\bar{x}\right)}^{2}}}$$
  where *CC* represents the correlation coefficient between IMF component and original signal, *r* represents the IMF, *x* represents the transformer vibration signal;
4. By setting the threshold *θ*, the corresponding IMF with larger *CC* is selected. If *CC* is greater than *θ*, the IMF will be kept as an effective component. Otherwise, the IMF will be abandoned as a useless part.
  (16) $$\theta =\sqrt{\frac{\sum_{i=1}^{n}{\left(CC_{i}-\bar{CC}\right)}^{2}}{n}}$$
  where *n* represents the number of IMFs;
5. The entropy values of extracted IMF components are calculated by MDE, and the entropy values formed into a characteristic matrix;
6. PCA is used to reduce the dimension of eigenvalue matrix;
7. After dimension reduction by PCA, the extracted feature vectors are sent to DPC to identify transformer faults.

**Figure 1:**
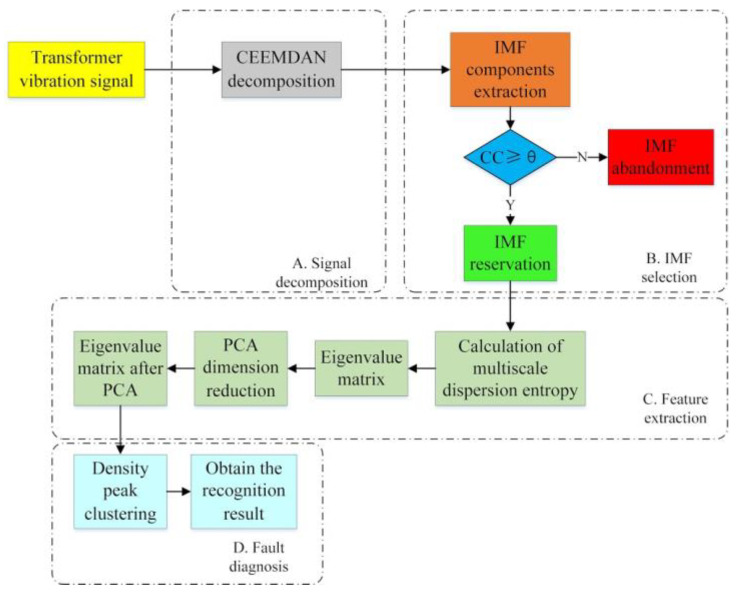
Block diagram of transformer vibration signal fault diagnosis based on CEEMDAN-MDE and DPC.

The block diagram of transformer vibration fault diagnosis based on CEEMDAN and MDE is shown in Figure 1.

## 3. Results Analysis

### 3.1. Simulation Analysis

**Figure 2:**
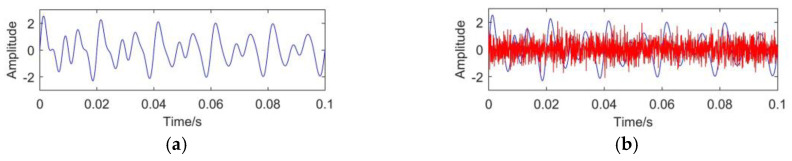
Simulated signal: (**a**) original signal; (**b**) noisy signal.

According to the characteristics of transformer vibration signals, a simulated vibration signal is designed to verify the effectiveness of CEEMDAN method. As defined in Equation (17), the original signal *s*(*t*) consists of three functions, namely, *f*_1_(*t*), *f*_2_(*t*), *f*_3_(*t*) with different amplitudes and frequencies. In on-site environment of transformers, the vibration signal is easily affected by the white noise. In order to simulate the real transformer vibration signal, the white noise is superimposed on the original signal. The white noise satisfies the Gauss distribution N (0, 0.6^2^). The final simulated signal *s*_1_(*t*) is described in Equation (18). The original signal and noisy signal are shown in Figure 2.

(17) $$\{\begin{array}{c} f_{1}(t)=\sin\left(20\pi t\right)\\ f_{2}(t)=\sin\left(30\pi t\right)\\ f_{3}(t)=e^{-2t}\sin\left(50\pi t\right)\\ s(t)=f_{1}(t)+f_{2}(t)+f_{3}(t) \end{array}$$

(18) $$s_{1}(t)=s(t)+\mathrm{normrnd}\left(0,0.6\right)$$

**Figure 3:**
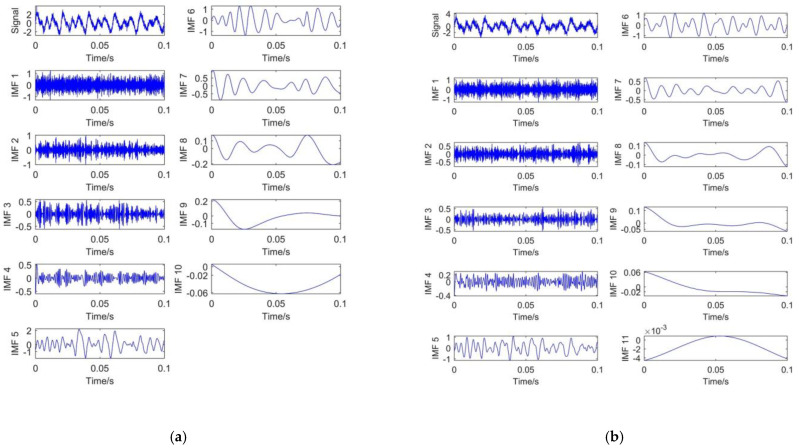

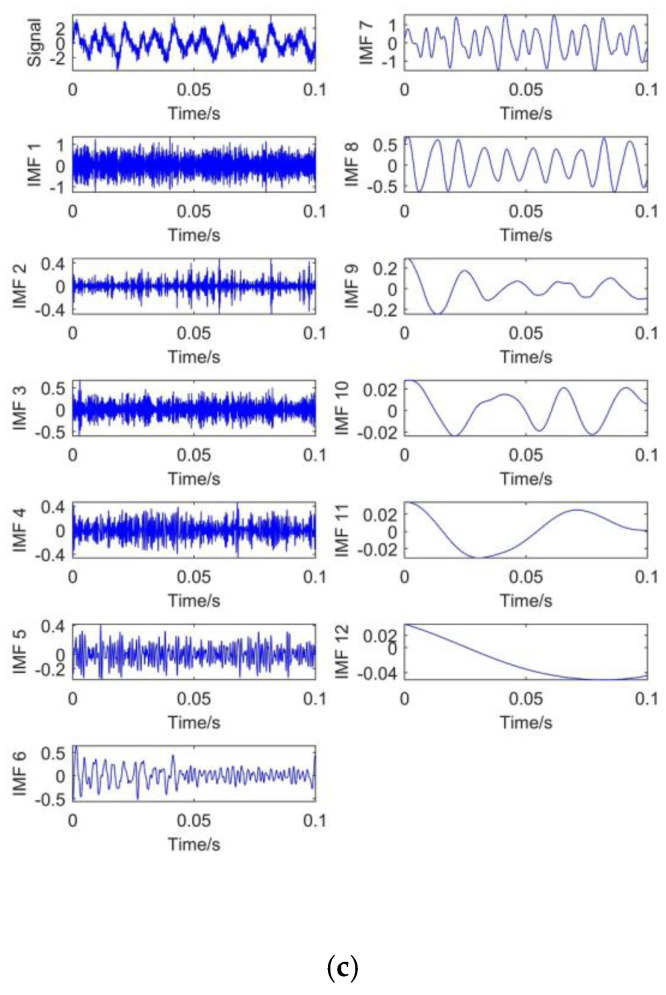
S_1_ signal decomposition: (**a**) EMD decomposition; (**b**) EEMD decomposition; (**c**) CEEMDAN decomposition.

To verify the effectiveness and superiority of CEEMDAN, EMD, and EEMD are also introduced to decompose the simulated signal. For EEMD and CEEMDAN, 100 groups of Gaussian white noise with standard deviation of 0.2 are added to the original signal. The decomposition results of different methods are shown in Figure 3.

Figure 3a presents the signal decomposition result based on EMD. It shows that 9 IMFs and a single residual component are obtained. The mode mixing phenomenon exists obviously in EMD decomposition.

Figure 3b is the decomposition result of EEMD. It can be seen that 10 IMFs and 1 residual component are obtained. There is also mode mixing phenomenon in EEMD decomposition. In addition, high frequency oscillation exists in IMF1, IMF2, and IMF3. It indicates that the added white noise is responsible for this phenomenon.

Figure 3c is the decomposition result of CEEMDAN. It shows that 11 IMFs and 1 residual component are obtained. It means that more details of the signal can be obtained through CEEMDAN. With the increase in IMF number, the corresponding frequency will decrease. Compared with EEMD, the frequency variation of CEEMDAN decomposition between different IMF components is more obvious. The reason is that CEEMDAN decomposition adds auxiliary noise into IMF components, rather than adding Gaussian white noise signal directly like EEMD. Moreover, EEMD uses the overall-averaging method for all modal components obtained from decomposition. Although CEEMDAN decomposition uses the overall-averaging calculation for the first-order IMF and repeat the above operation for the residual part. It can effectively solve the problem of noise transferring from high frequency to low frequency. Therefore, CEEMDAN decomposition can effectively avoid the of mode mixing phenomenon in EEMD.

From above figures, it can be found that CEEMDAN method is not only better than EMD and EEMD in suppressing mode mixing, but also more detailed in signal decomposition. It is convenient for subsequent signal processing. Generally, high frequency IMFs are dominated by noise, while low frequency IMFs are mainly composed of useful signals [35]. Therefore, the noisy parts and useful signal parts can be easily distinguished by CEEMDAN.

**Figure 4:**
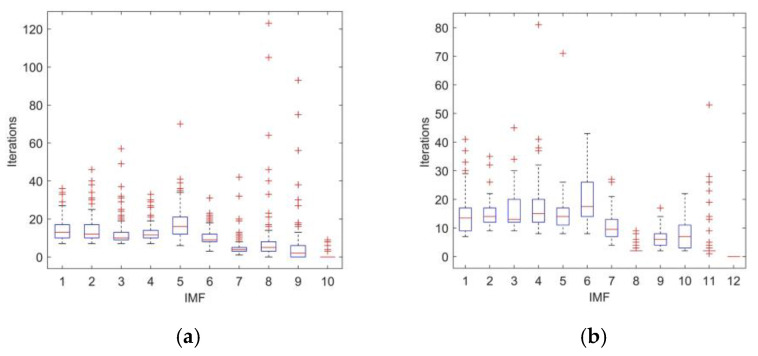
Iterations of IMF: (**a**) EEMD decomposition; (**b**) CEEMDAN decomposition.

Figure 4 shows the box diagram for calculating the number of IMF iterations using different decomposition methods.

In Figure 4, the ordinate is the number of iterations and the abscissa means the IMF components. The figure shows that for each IMF, the maximum number of iterations of EEMD method is 123, which is much larger than that of CEEMDAN. The computational complexity of CEEMDAN is nearly one third less than that of EEMD. This means that CEEMDAN can reduce the operation time and improve the efficiency of signal decomposition.

### 3.2. Experiment Analysis

In this paper, the vibration signals are obtained under experimental conditions. The vibration data of a SZ-20,000/35 three-phase winding transformer under three working conditions are collected. Firstly, the original signal is decomposed by CEEMDAN method and the IMF components are extracted. Secondly, the correlation coefficient method is applied to IMF components selection. Additionally, then the dispersion entropy value of each IMF component is calculated. Finally, PCA method is used to reduce the dimension of feature matrix. The extracted eigenvalues are used for pattern recognition.

#### 3.2.1. Signal Decomposition

**Figure 5:**
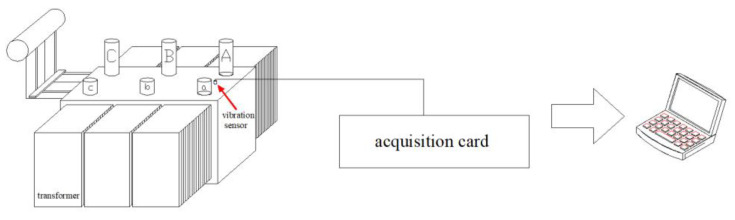
Experimental setup.

**Figure 6:**
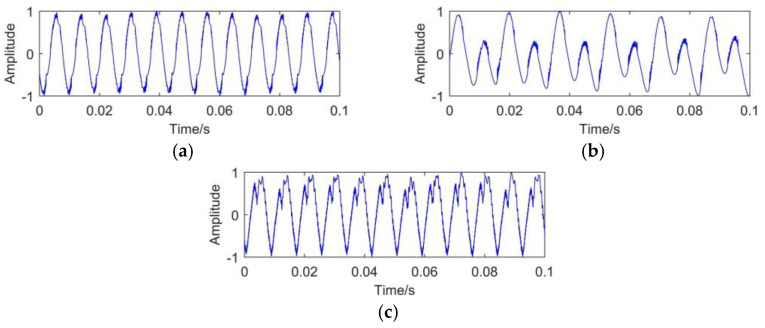
Transformer vibration signals under different working conditions: (**a**) NO; (**b**) WL; (**c**) CL.

Three kinds of transformer vibration signals are collected under different working conditions, which are normal operating conditions (NO), winding loosening (WL) and core loosening (CL), respectively. There are totally 90 groups of vibration signals, that is 30 groups for each working condition. In this paper, the piezoelectric acceleration sensor with sensitivity of 100 mV/g is used. The sampling frequency is set to 10 kHz. The transformer parameters are shown in Table 1. The experimental setup is shown in Figure 5. For convenient process and observation, the extracted vibration data are normalized. Transformer vibration signals under three different working conditions are shown in Figure 6.

**Figure 7:**
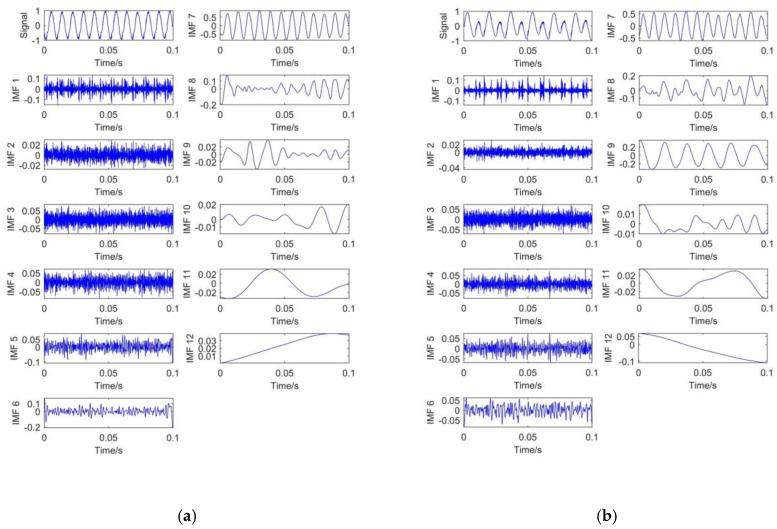

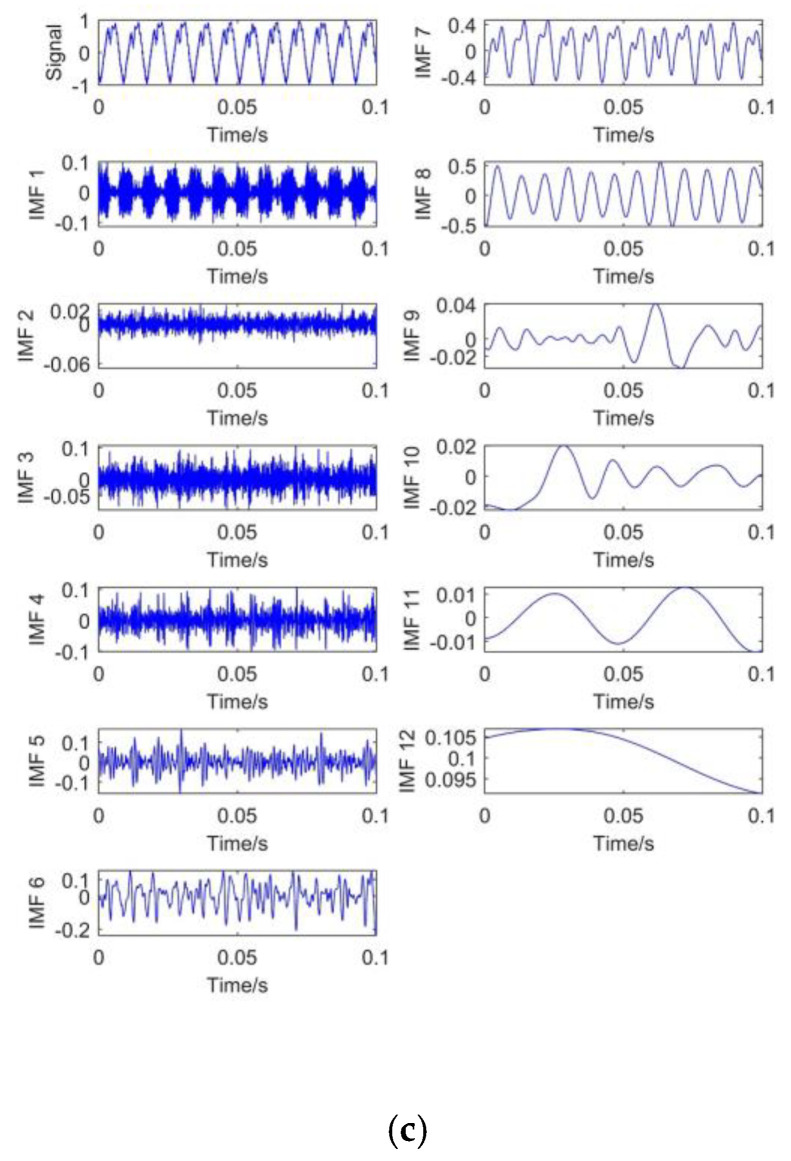
CEEMDAN decomposition: (**a**) NO; (**b**) WL; (**c**) CL.

Figure 6 shows that different vibration signals have different characteristics. In order to obtain the effective details of the vibration signal, this paper uses CEEMDAN to decompose the extracted signal. The decomposition results are shown in Figure 7.

Figure 7 shows that 11 IMF components and 1 residual component are obtained under each working condition. The IMF frequency is distributed from high frequency to low frequency. It can be seen from the high-frequency IMFs that there is no mode mixing phenomenon in this decomposition.

#### 3.2.2. IMF Component Extraction

**Figure 8:**
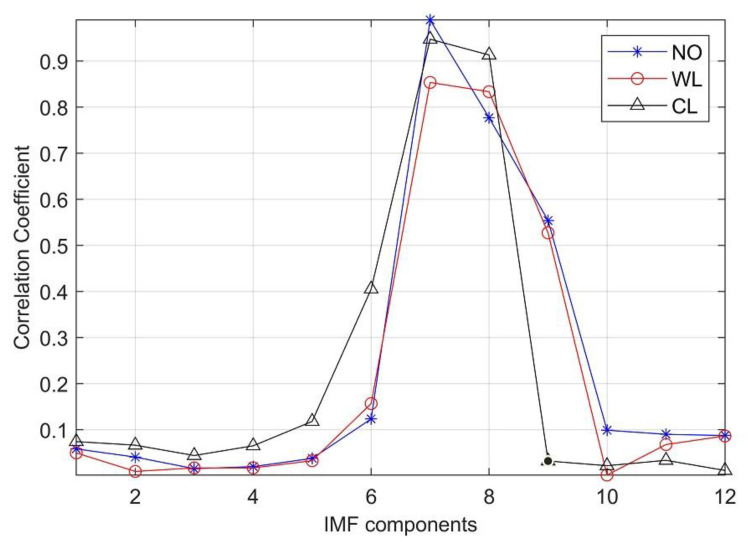
*CC* of IMF components.

Different types of transformer vibration signals have different characteristics. Therefore, effective information could be extracted from the vibration signal characteristics for faults recognition. In order to extract effective information of vibration signals, the correlation coefficient values between IMFs and the original signal are calculated. The IMF components with high correlation are selected as the effective component and others are abandoned as noisy parts. After several iterations and calculation, the *CC* results are shown in Figure 8.

As shown in Figure 8, different *CC* values are obtained varying with IMFs under three different conditions. The threshold can be defined according to Formula 16. After several calculation, the threshold of NO *θ*_1_ = 0.3214, the threshold value of WL *θ*_2_ = 0.3102 and the threshold of CL *θ*_3_ = 0.3298. After comparison with the threshold, the IMFs with larger *CC* can be reserved and those with smaller *CC* will be abandoned. Therefore, IMF7, IMF8 and IMF9 are selected as effective components for NO, IMF7, IMF8, and IMF9 for WL, and IMF6, IMF7, and IMF8 for CL.

#### 3.2.3. Entropy Calculation

Section 3.2.2 shows that effective IMFs are obtained by CEEMDAN decomposition and *CC* calculation. As is known, entropy is an effective tool for measurement of uncertainty. It has a good effect on non-stationary signals. In order to quantify the characteristic information of transformer vibration signal, MDE is introduced in this paper. To compare different performance of entropy, MSE and MPE are also introduced. After repeated trials, the parameters of MSE, MPE and MDE are shown in Table 2.

In Table 2, *m* is the embedding dimension, *r* is the matching threshold, *c* represents the class, *t* represents the time delay, * represents NULL. The scale selected in this paper is set to be 10. After multiple calculation, the entropy values of the selected IMFs are obtained shown as eigenvalue matrixes in the following part. The eigenvalue matrix formed by MDE are present as follows. In which *M*_1_ means the values obtained in the NO, *M*_2_ means the values obtained in the WL condition and *M*_3_ means the values obtained in the CL.

$$M_{1}=\left(\begin{array}{c} 2.1853\;2.4740\;2.6681\;2.7988\;2.8372\;2.9020\;2.9235\;2.8719\;2.9718\;2.8629\\ 2.2251\;2.5271\;2.7576\;2.9374\;3.1009\;3.2228\;3.2919\;3.4188\;3.4087\;3.4631\\ 2.1612\;2.4146\;2.6110\;2.7765\;2.9087\;3.0295\;3.1070\;3.1653\;3.2341\;3.3445 \end{array}\right)$$

$$M_{2}=\left(\begin{array}{c} 2.1981\;2.4870\;2.6891\;2.8200\;2.9001\;2.9554\;3.0079\;3.0125\;3.0232\;3.1618\\ 2.1573\;2.4243\;2.6284\;2.7979\;2.9562\;3.0624\;3.1600\;3.2035\;3.2805\;3.4215\\ 1.9838\;2.1685\;2.3219\;2.4491\;2.5652\;2.6440\;2.7335\;2.7908\;2.8441\;2.8927 \end{array}\right)$$

$$M_{3}=\left(\begin{array}{c} 2.7998\;3.3170\;3.6506\;3.9145\;4.0896\;4.3102\;4.4191\;4.3861\;4.4807\;4.4675\\ 2.3714\;2.7221\;2.9785\;3.1481\;3.2685\;3.2854\;3.3819\;3.4312\;3.5245\;3.6284\\ 2.2042\;2.4916\;2.6987\;2.8476\;2.9545\;3.0310\;3.0440\;3.1080\;3.2110\;3.2117 \end{array}\right)$$

The eigenvalue matrixes obtained by MDE calculation show that each matrix is multidimensional, and the correlation between each eigenvalue tends to be high. It is not convenient for subsequent processing. Therefore, it is necessary to reduce the dimension of the eigenvalue matrixes. In this paper PCA method is introduced to realize the dimension reduction.

#### 3.2.4. Dimension Reduction

**Figure 9:**
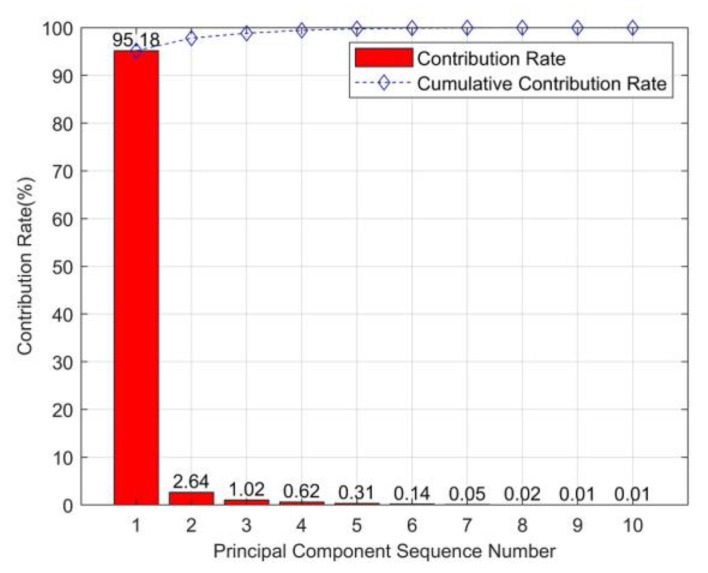
Principal component contribution rate.

In order to extract useful information, PCA is employed to reduce the dimension of entropy values. In this part, the eigenvalue matrix is defined as $M=\left\{P^{T},Q^{T},R^{T}\right\}$. The vector *P* in the matrix *M*_1_ is taken as an example. The contribution varying with principal components by PCA method is shown in Figure 9.

**Figure 10:**
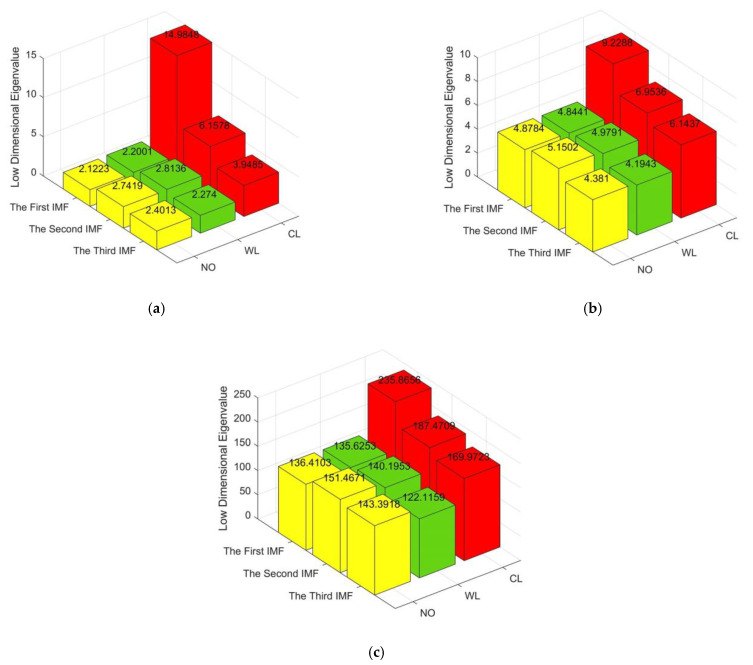
Eigenvalue matrixes: (**a**) MSE; (**b**) MPE; (**c**) MDE.

Figure 9 shows that the contribution rate varies with different principal components. The contribution rate of the first principal component is 95.18% which represents the dominant information of the data sequence. Therefore, the first principal component can be selected as the useful eigenvector. The other components with small contribution rates can be abandoned. Similarly, the contribution rate of vector *Q* and *R* in matrix *M*_1_ can be obtained. Through PCA dimension reduction, the 3 × 10 eigenvalue matrix formed in Section 3.2.3 is effectively simplified as 3 × 1 matrix. With PCA, the eigenvalue matrixes under three conditions formed by MSE, MPE and MDE are present in Figure 10.

Figure 10 indicates that the simplified eigenvalue matrixes are obtained through PCA method, which can greatly improve the operation speed. It can not only reduce the number of eigenvalues, but also preserve the strongly correlated eigenvalues. Therefore, the dimension reduction in eigenvalue matrixes reduces the burden of identification.

#### 3.2.5. Clustering and Recognition

**Figure 11:**
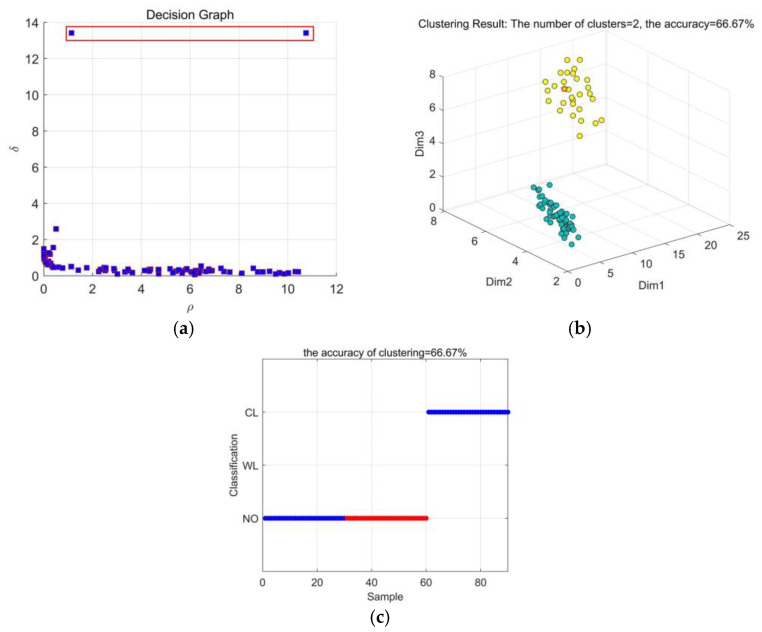
Results based on CEEMDAN-MSE: (**a**) Decision graph; (**b**) DPC clustering effect; (**c**) Recognition results.

**Figure 12:**
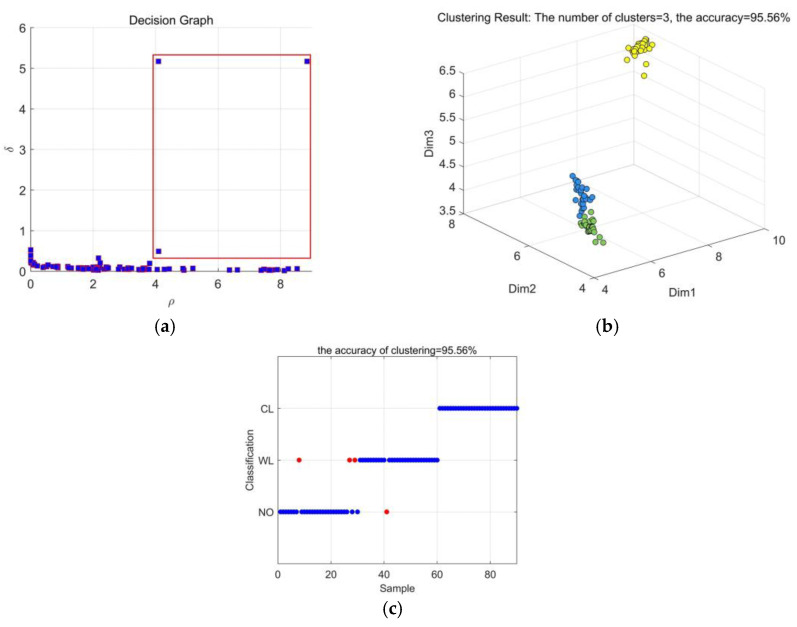
Results based on CEEMDAN-MPE: (**a**) Decision graph; (**b**) DPC clustering effect; (**c**) Recognition results.

**Figure 13:**
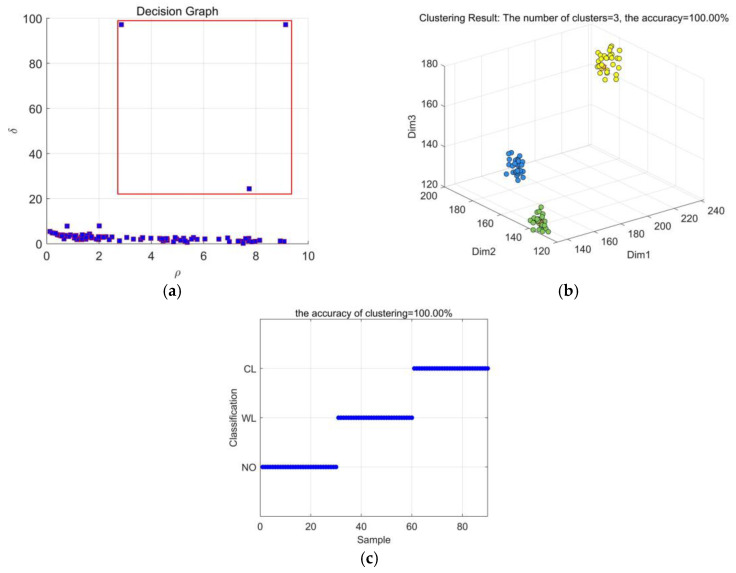
Results based on CEEMDAN-MDE: (**a**) Decision graph; (**b**) DPC clustering effect; (**c**) Recognition results.

As described above, each type of transformer vibration signal can be eventually present as a three-dimensional matrix. In this paper, density peaks clustering (DPC) is introduced to discriminate different types of transformers vibration faults with extracted feature matrix. DPC is an unsupervised method with no need for training samples. It can find the correct number of clusters without pre-setting. In this work, 30 groups of vibration signals for each working condition are collected. Using different feature extraction approaches, the diagnostic results of DPC method are shown in Figure 11, Figure 12 and Figure 13.

Figure 11 presents the DPC result based on CEEMDAN-MSE. From Figure 11a, it can be found that only 2 kinds of decision points can be selected. It is difficult to cluster accurately by using the finite decision points. Figure 11b shows that only 2 types of working conditions can be distinguished clearly. Figure 11c shows that the recognition accuracy of CEEMDAN-MSE is 66.67%. The type of WL cannot be detected.

From Figure 12a, it can be found that 3 decision points can be selected in the decision graph. However, Figure 12b shows that 2 types of vibration signals mix with each other. It is difficult to distinguish between NO and WL type. Figure 12c shows that the recognition accuracy of CEEMDAN-MPE through DPC clustering is 95.56%. However, there are still some misjudges in the recognition. Three NO type vibration signals are misjudged as WL. One WL type vibration signal is misjudged as NO type. It indicates that the difference between NO and WL type is not easy to distinguish.

Figure 13a shows that three decision points can be selected in the decision graph. From Figure 13b, it can be seen that 3 types of working conditions can be distinguished clearly. Figure 13c shows that the recognition accuracy of CEEMDAN-MDE through DPC clustering is 100%. The method used in this paper is not only accurate to select decision points, but also has good recognition effect. It also shows that MDE is better than the MSE and MPE in distinguishing different groups of each data set. MDE can overcome the problems of equal embedded vector values and discarding some information about amplitude in MPE algorithm, and MDE is very sensitive to the change of frequency, amplitude, and bandwidth. Therefore, MDE is suitable for feature extraction of transformer vibration signal.

**Figure 14:**
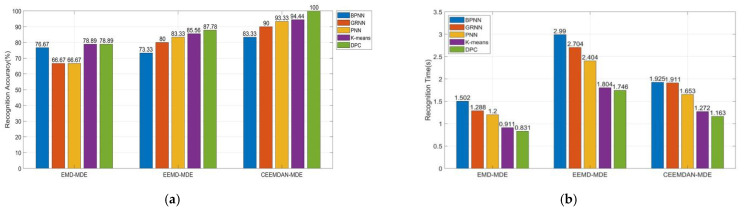
Recognition results: (**a**) Recognition accuracy; (**b**) Recognition time.

In order to verify the superiority of DPC method, different approaches are introduced in this paper, including K-means, BPNN, GRNN and PNN. With the same vibration signal samples described above, these methods are employed for fault recognition. After several times of trials, the parameters of BPNN, GRNN, PNN are present in Table 3. Moreover, to verify the superiority of the proposed method, EMD and EEMD are introduced. The recognition accuracy and time of different methods are shown in Figure 14.

In Table 3, * represents NULL.

Figure 14 shows the recognition accuracy and recognition time of EMD, EEMD, and CEEMDAN. It can be concluded from Figure 14a that different feature extraction methods show diverse performance in the recognition accuracy. The feature extraction based on CEEMDAN-MDE combines the advantages of CEEMDAN and MDE. Compared with other two feature extraction approaches, it gets better diagnostic results. Moreover, different algorithms are employed for transformer vibration signal recognition. After comparison, DPC clustering gets the best performance in the diagnostic accuracy, with 100% recognition rate. DPC clustering can automatically find the correct number of clusters according to the local maximum of data point density. K-means method achieves good performance in the recognition, but it has some inherent restriction in practice. It depends on the initial given number of clusters, and random initialization may generate different clustering effects. The recognition accuracy of BPNN, PNN, and GRNN is unsatisfying. The supervised learning needs a large number of training samples. The recognition accuracy of supervised learning methods is not high enough in the case of small samples.

By observing the recognition time of various recognition methods in Figure 14b, it shows that EMD has the shortest recognition time. However, the recognition accuracy of EMD is much lower than other methods. The reason is that EMD decomposition signal does not need Gaussian white noise which can effectively reduce the operation time. However, the inherent mode mixing problem of EMD has an adverse effect on the recognition accuracy. As unsupervised learning does not need training samples, the time of unsupervised clustering learning is obviously shorter than that of supervised learning methods.

In general, the combination of CEEMDAN and MDE can effectively extract the dominant features of transformer vibration signals. Additionally, the recognition effect of DPC is better than other recognition methods. Therefore, CEEMDAN-MDE-DPC is feasible for transformer vibration fault diagnosis.

To test the sensitivity to noise of the proposed method, the diagnostic results are present before and after de-noising. Many studies have proved the de-noising effectiveness of wavelet method in non-stationary signals [36,37]. In this work, wavelet method is used for transformer vibration signal de-noising with heursure soft threshold. The results of different recognition methods based on CEEMDAN-MDE before and after wavelet de-noising are shown in Table 4.

Table 4 indicates that after de-noising, the improvement in recognition accuracy of different recognition methods is not clear enough. Moreover, no changes have occurred for the recognition accuracy of PNN and DPC algorithms before and after denoising. Therefore, the proposed feature extraction method is insensitive to the noise. This may greatly improve the diagnostic efficiency in practice.

## 4. Conclusions

Power transformer is the pivotal equipment in the power system. Transformer fault diagnosis has a very high value in practice. This paper presents a fault diagnosis method which combines the advantages of CEEMDAN, MDE, and DPC. It uses a combination of CEEMDAN and MDE to extract the characteristics of transformer vibration signals. Afterwards, this work uses DPC for fault identification. The conclusions are as follows:

1. CEEMDAN method can effectively solve the problem of mode mixing in EMD and EEMD. The signal decomposed by CEEMDAN is more detailed. Moreover, the computational complexity of CEEMDAN is less than that of EEMD. It can effectively reduce the operation time and improve the efficiency of signal decomposition. By comparing the results before and after signal denoising, it also shows that CEEMDAN has good anti-noise ability;
2. Entropy effectively quantifies the complexity and uncertainty of time series. MDE can overcome the problems of information loss in MPE and slow speed for long signal applications in MSE. In addition, it is very sensitive to the changes in the simultaneous frequency, amplitude and bandwidth of the signal. From the recognition results in this paper, it can be seen that MDE can effectively quantitate the characteristics of power transformer vibration signals;
3. DPC is an effective tool for pattern recognition. DPC is able to detect non-spherical clusters and automatically find the correct number of clusters. As an unsupervised algorithm, it does not need training samples, which can effectively reduce the operation time. Experimental results show that DPC can accurately identify the types of transformer vibration signals with fewer samples. Compared with K-means, BPNN, GRNN and PNN, DPC gets higher recognition accuracy.

In conclusion, the combination of CEEMDAN, MDE and DPC proposed in this paper can extract effective vibration signal information, and accurately distinguish different types of transformers faults. It provides an effective tool for transformer fault diagnosis. Considering that different measurement circuits and sensors may cause different characteristics of transformer vibration signals, future attempts will be made to extract signals from different measurement conditions to verify the effectiveness of the proposed method.

## Figures and Tables

**Table 1:** Transformer parameters.

Parameter Category	Parameter Value
Rated Capacity	20,000 kVA
Rated Voltage	35 ± 3 × 2.5%/10 kV
No-Load Current	0.43%
Impedance Voltage	8%

**Table 2:** Parameter setting.

	MSE	MPE	MDE
*m*	2	3	3
*r*	0.2	*	*
*c*	*	*	6
*t*	*	*	1

**Table 3:** Parameter setting.

Classifier	Number of Neurons in Input Layer	Number of Neurons in Hidden Layer	Number of Neurons in Output Layer
BPNN	3	3	3
GRNN	3	*	3
PNN	3	*	3

**Table 4:** Recognition accuracy.

Method	Results without De-Noising (%)	Results with De-Noising (%)
BPNN	83.33	83.33
GRNN	90.00	93.33
PNN	93.33	93.33
K-Means	94.44	100.00
DPC	100.00	100.00

## Data Availability

The raw data required to reproduce these findings cannot be shared at this time as the data also forms part of an ongoing study.
